# Association of 71 cardiovascular disease-related plasma proteins with pulmonary function in the community

**DOI:** 10.1371/journal.pone.0266523

**Published:** 2022-04-07

**Authors:** Jenna N. McNeill, Dong Heon Lee, Shih-Jen Hwang, Paul Courchesne, Chen Yao, Tianxiao Huan, Roby Joehanes, George T. O’Connor, Jennifer E. Ho, Daniel Levy

**Affiliations:** 1 Division of Pulmonary and Critical Care, Massachusetts General Hospital, Boston, Massachusetts, United States of America; 2 The Framingham Heart Study, Framingham, Massachusetts, and the Population Sciences Branch, Division of Intramural Research, National Heart, Lung, and Blood Institute, National Institutes of Health, Bethesda, Maryland, United States of America; 3 Pulmonary Center, Boston University, Boston, Massachusetts, United States of America; 4 Division of Cardiology, Beth Israel Deaconess Medical Center, Boston, Massachusetts, United States of America; Medizinische Universitat Graz, AUSTRIA

## Abstract

**Rationale:**

It has been speculated that shared mechanisms underlie respiratory and cardiovascular diseases (CVD) including systemic inflammation or mutual risk factors. In this context, we sought to examine the associations of CVD-related plasma proteins with lung function as measured by spirometry in a large community-based cohort of adults.

**Methods:**

The study included 5777 Framingham Heart Study participants who had spirometry and measurement of 71 CVD-related plasma proteins. The association of plasma proteins with lung function was assessed cross-sectionally and longitudinally using models accounting for familial correlations. Linear mixed models were used for the following measurements: FEV_1%predicted_, FVC_%predicted_, and FEV_1_/FVC ratio with secondary analyses examining obstructive and restrictive physiology at baseline and their new onset during follow up.

**Measurements and main results:**

Among the 71 CVD-related plasma proteins, 13 proteins were associated in cross-sectional analyses with FEV_1%predicted_, 17 proteins were associated with FVC_%predicted_, and 1 protein was associated with FEV_1_/FVC. The proteins with the greatest inverse relations to FEV_1%predicted_ and FVC_%predicted_ included leptin, adrenomedullin, and plasminogen activator inhibitor-1; in contrast there were three proteins with positive relations to FEV_1%predicted_ and FVC_%predicted_ including insulin growth factor binding protein 2, tetranectin, and soluble receptor for advanced glycation end products. In longitudinal analyses, three proteins were associated with longitudinal change in FEV_1_ (ΔFEV_1_) and four with ΔFVC; no proteins were associated with ΔFEV_1_/FVC.

**Conclusion:**

Our findings highlight CVD-related plasma proteins that are associated with lung function including markers of inflammation, adiposity, and fibrosis, representing proteins that may contribute both to respiratory and CVD risk.

## Introduction

Heart disease and respiratory disease are the first and fourth leading causes of mortality in the United States [1, 2]. A complex interplay between the pulmonary and cardiac systems with respect to clinical outcomes has long been recognized [3]. For example, a decline in pulmonary function has been associated with greater risk for cardiovascular disease (CVD) after accounting for CVD risk factors [3, 4]. The presence of chronic obstructive pulmonary disease (COPD) has been reported to be associated with increased odds of developing CVD by nearly three-fold, while idiopathic pulmonary fibrosis (IPF) has been linked to fourfold increased odds of multi-vessel coronary artery disease (CAD) [5, 6]. Conversely, heart failure has been reported to be associated with lower FEV_1_ and FVC after accounting for smoking and body size [7, 8]. The close link between pulmonary and cardiac diseases and the interaction between them that culminates in adverse outcomes remains incompletely understood. It is also apparent that there are shared risk factors (e.g. cigarette smoking) and biological pathways (e.g. systemic inflammation leading to oxidative stress and endothelial and alveolar damage) underlying both diseases [9, 10].

We hypothesized that CVD-related proteins are also associated with pulmonary function and lung disease [11]. To this end, we sought to examine the associations of 71 CVD-related plasma proteins with lung function measured by spirometry both cross-sectionally and longitudinally in a large community-based cohort of adults. Our goal was to identify protein biomarkers associated with pulmonary function that would provide insights into the association of lung disease and CVD.

## Methods

### Study sample

The baseline sample for cross-sectional analyses consisted of Framingham Heart Study (FHS) participants who attended Offspring cohort examination 7 (year 1995–1999, n = 3539) and Third Generation (Gen 3) cohort examination 1 (year 2002–2005, n = 4095) [12, 13]. Participants had to have a biosample for measurement of plasma proteins and spirometry measurement of pulmonary function (Offspring n = 2282, Gen 3 n = 3613). We excluded individuals with prevalent heart failure (Offspring cohort n = 16, Gen 3 n = 2), chronic kidney disease defined as an estimated glomerular filtration rate (eGFR) <30 ml/min/1.73m^2^ (Offspring n = 5), asthma (identified through medication usage and self-reported diagnosis n = 256), those with alpha-1 antitrypsin deficiency genotype (n = 9), and individuals missing key covariates (n = 136), leaving 5777 participants for analysis. All participants gave written informed consent. The study was approved by the Boston Medical Center Institutional Review Board. For longitudinal analyses, participants who did not attend the follow-up clinical examination and provide spirometry data (N = 267), those with obstructive (Category 1 N = 489, Category 2 N = 343) or restrictive physiology (N = 201) at baseline were excluded.

### Clinical assessment and covariates

At the baseline examination, a medical history, physical examination, and laboratory testing were collected. Information on cigarette smoking was based on self-report. Other covariates included BMI (kg/m^2^) and diabetes mellitus, which was defined as a fasting glucose ≥126 mg/dL or the use of hypoglycemic medications for treating hyperglycemia [11]. A history of CVD including myocardial infarction, angina pectoris, coronary insufficiency, cerebrovascular accident, atherothrombotic infarction of brain, transient ischemic attack, cerebral embolism, intracerebral hemorrhage, subarachnoid hemorrhage, intermittent claudication, or congestive heart failure was abstracted from relevant medical records, hospital records, electrocardiograms, and/or cardiac enzymes. Each diagnosis was verified by a three-physician review panel.

### Measurements of plasma proteins

The Systems Approach to Biomarker Research (SABRe) in CVD initiative was created by the NHLBI to identify biomarkers related to CVD and associated risk factors [11]. A platform of 85 plasma proteins were selected based on comprehensive literature review, gene expression profiling and genome-wide association studies of myocardial infarction or coronary heart disease within the FHS cohort and others [11]. The proteins were measured by Sigma Aldrich, Inc (St. Louis, MO) using the Luminex xMAP platform (Luminex, Inc., Austin, TX). 71 of the 85 biomarkers were included in this analysis given detectable levels for >95% of the participants. Among the 71 biomarkers utilized <2% missing values [11]. The mean coefficient of variation across the 71 proteins was 2.2% with a range of 0 to 17.1% as previously described by Ho et al., 2018 [11].

### Measurements of pulmonary function

All 5777 participants underwent spirometry testing at the baseline examination using the Collins survey II Water-Seal spirometer (Warren Collins, Inc., Braintree, MA, USA) and acquisition and quality control software (S&M Instruments, Doylestown, PA, USA) at the 7^th^ examination of the Offspring cohort (1998–2001), and first examination of the Third Generation cohort (2002–2005). A total of 4477 participants (1636 Offspring at Exam 8 (2005–2008) and 2841 Third Generation at Exam 2 (2008–2011) underwent repeat assessments of spirometry at a subsequent examination using the Collins Comprehensive Pulmonary Laboratory system (nSpire Health Inc., Longmont, CO, USA) [14]. Continuous measures of pulmonary function (FEV_1%predicted_, FVC_%predicted,_ FEV_1_/FVC) were calculated using the published reference values and equations derived from the NHANES III study [15].

Restrictive lung physiology (RLP) was defined as FVC <80_% predicted_ and FEV_1_/FVC >0.7 [16]. Obstructive lung physiology was defined based on the Global Initiative for Chronic Obstructive Lung Disease (GOLD) COPD definitions [17]. Individuals with FEV_1_/FVC <0.7 and 80%≤FEV_1%predicted_ ≤100% predicted were categorized as having Category 1 COPD, and those with FEV_1_/FVC <0.7 and FEV_1%predicted_ <80% were categorized as Category 2 COPD.

Based upon the GOLD criteria we had 21 participants who met criteria for GOLD 3, (FEV1% predicted 30–49%) and no participants who met criteria for GOLD 4 (FEV_1_% predicted less than <30%). Those who met criteria for GOLD 3 and GOLD 4 were included in category 2 COPD given the small numbers.

### Statistical analysis

Protein concentrations were rank-normalized for analysis due to right-skewed distributions. We examined the association of individual plasma proteins with lung function measures using generalized linear mixed models (GLMM) to adjust for familial correlations. Linear mixed models were used for the following measurements: FEV_1%predicted_, FVC_%predicted_, FEV_1_/FVC (all primary outcomes), with secondary analyses examining longitudinal changes in spirometry traits (ΔFEV_1%predicted_/year, ΔFVC_%predicted_/year, ΔFEV_1_/FVC/year) among individuals with serial spirometry assessments. Models were adjusted for age, sex, body mass index (BMI), smoking status (yes = current or former, no = never), pack-years of cigarette smoking, and diabetes mellitus (yes/no). For longitudinal change analyses, we adjusted for the pulmonary function trait at baseline. We used logistic regression models using generalized estimating equations to assess the association of biomarkers with dichotomous outcomes of obstructive physiology and restrictive physiology. We defined a significant p-value cut-point of 7.04E-04 (0.05/71; 71 proteins included in analysis). In addition, to evaluate model discrimination as assessed by the c-statistic, biomarker that were identified as having a cross sectional association with restrictive or obstructive physiology, as defined by p<0.05, were added to the model containing clinical covariates [18].

For the FEV_1%predicted_ or FVC_%predicted_ associated protein biomarkers, we analyzed linear regression tests separately for smokers and non-smokers. For the secondary analyses, we applied quintile rank value referring to individual protein measurement to generate least square means and 95% confidences intervals for the lung function measurements, FEV_1%predicted_, FVC_%predicted_. Results of the least square mean calculations were applied to provide graphic representation of the linear associations.

In exploratory analyses, we repeated the linear mixed models for dependent traits of FEV_1%predicted_ or FVC_%predicted,_ by adding an interaction term of smoking and the normalized protein values. We assessed the statistical significance of smoking vs. biomarker interaction with a p-value threshold of 0.05. All analyses were conducted using SAS version 9.4.

## Results

A total of 5777 participants were included in the cross-sectional protein-trait analysis (mean 48 ± 13 years, 53% women, 14% current smokers and 37% former smokers; Table 1). The average BMI was 27.4±5.4 kg/m^2^, 6% had diabetes mellitus, and 4% had CVD at baseline. The majority of participants had normal lung function with mean FEV_1%predicted_ of 97 ±14, FVC_%predicted_ of 101±12 and FEV_1_/FVC of 96 ±8. At baseline, 14% of participants met criteria for obstructive lung physiology (8% category 1 and 6% category 2) and 3% met criteria for restrictive lung physiology.

**Table 1:** Clinical characteristics of 5777 FHS participants at baseline.

	ALL (N = 5777)	Normal (N = 4744)	Restrictive (N = 201)	Obstructive Category 1 (N = 489)	Obstructive Category 2 (N = 343)
Age, years, mean (SD)years*	48 (13)	46 (13)	55 (12)	54 (13)	59 (12)
Women N (%)	3089 (54%)	2577 (54%)	106 (53%)	236 (48%)	170 (50%)
Current smoker, N (%)	808 (14%)	590 (12%)	33 (16%)	82 (17%)	103 (30%)
Former smoker, N (%)	2125 (37%)	1654 (35%)	86 (43%)	278 (57%)	160 (47%)
Pack-years smoking, mean (SD)	10 (16)	7 (13)	16 (21)	16 (22)	29 (26)
Body-mass-index (kg/m^2^, mean (SD)	27.4 (5.4)	27.2 (5.3)	31.1 (7.1)	26.4 (4.3)	28.4 (5.6)
Diabetes Mellitus, N, (%)	326 (6%)	213 (4%)	38 (19%)	32 (7%)	43 (13%)
FEV_1% predicted_ (%,median (Q1,Q3))	97 (89,106))	100 (93,108)	76 (72,80)	89 (84,94)	72 (64,76)
FVC_%predicted_ (%,median (Q1,Q3))	101 (93,109)	102 (95,110)	76 (73,79)	105 (99,111)	86 (79,92)
FEV_1_/FVC _% predicted_ (%,median (Q1,Q3))	77(73,81) (8)	78 (75,81)	67 (65,69)	67 (65,69)	63 (58,67)

Restrictive was defined as FVC < 80% predicted and FEV_1_/FVC>0.7.

Obstructive Category 1 was defined as FEV_1_/FVC <0.7 and 80%≤FEV_1_ ≤100% predicted.

Obstructive Category 2 was defined as FEV_1_/FVC <0.7 and FEV_1_ <80% predicted.

*Anova test for differences in age across the four groups revealed p<1.0E-22.

### Cross-sectional analysis of proteins associated with pulmonary function measures

**Fig 1:**
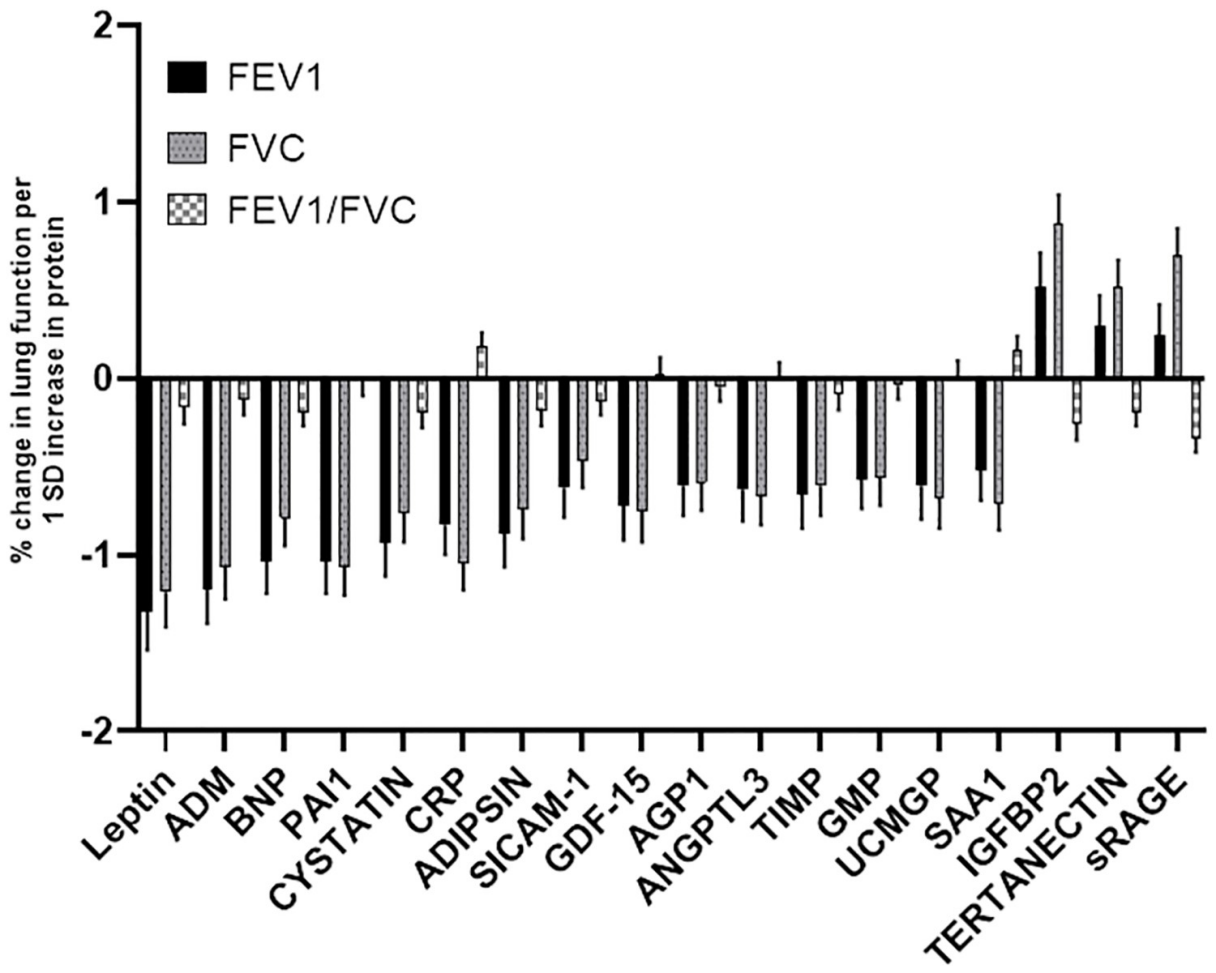
Cross-sectional association of cardiovascular disease related plasma proteins with lung function. There were 18 proteins with associations with baseline FEV_1%predicted_, FVC_%predicted,_ or FEV_1_/FVC ratio. Of these proteins, 15 were associated with lower FEV_1%predicted_ and FVC_%predicted_, with little effect of FEV_1_/FVC ratio.

Among the 71 CVD-related plasma proteins, 13 proteins were associated (all inversely) with FEV_1%predicted_, including proteins representing adipokine and inflammatory markers (Fig 1, Table 2, S2 Table). The strongest associations were with leptin (P = 5.85E-10), adrenomedullin (ADM; (P = 1.98E-09), and plasminogen activator inhibitor-1 (PAI1; P = 1.25E-8).

**Table 2:** Cross-sectional associations of proteins at baseline with FEV_1%predicted_, FVC_%predicted_, and FEV_1_/FVC.

	FEV_1%predicted_			FVC_%predicted_			% FEV1/FVC		
	Beta	SE	p-value	Beta	SE	p-value	Beta	SE	p-value
LEPTIN	**-1.36**	**0.22**	**5.85E-10**	**-1.22**	**0.20**	**5.99E-10**	-0.19	0.10	6.55E-02
ADM	**-1.20**	**0.20**	**1.98E-09**	**-1.05**	**0.18**	**3.58E-09**	-0.14	0.09	1.33E-01
PAI1	**-1.06**	**0.19**	**1.25E-08**	**-1.09**	**0.17**	**5.03E-11**	-0.01	0.09	8.74E-01
BNP	**-1.01**	**0.18**	**2.57E-08**	**-0.74**	**0.16**	**4.32E-06**	-0.21	0.08	1.29E-02
CYSTATIN C	**-0.97**	**0.19**	**2.56E-07**	**-0.75**	**0.17**	**7.90E-06**	-0.22	0.09	1.26E-02
ADIPSIN	**-0.92**	**0.19**	**1.26E-06**	**-0.73**	**0.17**	**1.70E-05**	-0.22	0.09	1.17E-02
CRP	**-0.84**	**0.17**	**1.68E-06**	**-1.06**	**0.16**	**9.25E-12**	0.18	0.08	2.50E-02
AGP1	**-0.66**	**0.18**	**1.78E-04**	**-0.62**	**0.16**	**7.81E-05**	-0.06	0.08	4.70E-01
SICAM1	**-0.64**	**0.17**	**2.19E-04**	-0.47	0.15	2.08E-03	-0.14	0.08	8.12E-02
TIMP1	**-0.71**	**0.19**	**2.35E-04**	**-0.63**	**0.17**	**2.72E-04**	-0.11	0.09	2.23E-01
GDF15	**-0.75**	**0.20**	**2.53E-04**	**-0.77**	**0.18**	**2.57E-05**	0.01	0.10	9.02E-01
GMP140	**-0.60**	**0.17**	**5.67E-04**	**-0.60**	**0.16**	**1.27E-04**	-0.04	0.08	6.28E-01
ANGPTL3	**-0.62**	**0.18**	**6.72E-04**	**-0.66**	**0.16**	**5.13E-05**	0.01	0.09	9.16E-01
UCMGP	-0.60	0.19	1.71E-03	**-0.62**	**0.17**	**2.38E-04**	-0.01	0.09	8.68E-01
SAA1	-0.55	0.17	1.24E-03	**-0.75**	**0.15**	**5.78E-07**	0.17	0.08	3.51E-02
IGFBP2	0.50	0.19	7.01E-03	**0.90**	**0.17**	**5.63E-08**	-0.29	0.09	8.50E-04
TERTANECTIN	0.28	0.17	9.12E-02	**0.54**	**0.15**	**2.91E-04**	-0.22	0.08	5.01E-03
sRAGE	0.24	0.18	1.67E-01	**0.70**	**0.16**	**8.86E-06**	**-0.35**	**0.08**	**2.41E-05**

Beta coefficient represents linear correlation between lung function measurement and rank normalized protein. MV model adjusted for age, sex, body mass index (BMI), smoking status (yes = current/former, no = never), pack-years of cigarette smoking, and diabetes mellitus (yes/no).

*Bolded numbers represent statistically significant values meeting Bonferroni corrected p-value threshold: 0.05/71 = 7.04E-04.

Seventeen proteins were associated with FVC_%predicted_ (Fig 1, Table 2); the top proteins were C-reactive protein (CRP; P = 9.25E-12), PAI-1 (P = 5.03E-11), and leptin (P = 5.99E-10). Of these 17 proteins, 12 were also associated with FEV_1%predicted_, and one (sRAGE) with FEV_1_/FVC. Proteins that were associated with both FEV_1%predicted_ and FVC_%predicted_ included adipokines (leptin, adipsin), inflammatory proteins (ADM, CRP, PAI-1), and the cardiovascular-related proteins (growth differentiation factor-15 (GDF-15), B-type natriuretic protein (BNP)). Of note, among the plasma proteins associated with spirometry traits, the majority demonstrated a negative association (i.e., higher protein level was associated with lower spirometry value). There were three proteins showing a positive association with FVC_%predicted_: IGFBP2 (β = 0.90, s.e. = 0.17, P = 5.63E-08), tetranectin (β = 0.54, s.e. = 0.15, P = 2.91E-04) and sRAGE (β = 0.70, s.e. = 0.16, P = 8.86E-06) (Table 2). The only protein associated with FEV_1_/FVC cross sectionally was sRAGE (β = -0.35, s.e. = 0.08, P = 2.41E-05) (Table 2).

When examining the cross-sectional association of proteins with dichotomous lung function traits including restrictive and obstructive physiology, we found no significant associations at the Bonferroni-corrected p-value threshold (results for nominal P<0.05) and minor changes in c-statistic with the addition of individual proteins on top of the clinical model (Table 3).

**Table 3:** Cross-sectional association of cardiovascular disease related plasma proteins with restrictive and obstructive physiology.

Restrictive Physiology N = 201			
Protein	OR (95% CI)	p-value	C-statistic
**Baseline clinic model**			**0.762**
ADM	1.24 (1.08–1.42)	1.74E-03	0.771
SAA1	1.16 (1.05–1.29)	4.29E-03	0.768
AGP1	1.21 (1.06–1.39)	5.13E-03	0.767
CRP	1.14 (1.03–1.26)	8.62E-03	0.776
LEPTIN	1.17 (1.03–1.32)	1.52E-02	0.767
FBN	1.13 (1.02–1.24)	1.71E-02	0.767
CYSTATIN-C	1.18 (1.02–1.36)	2.44E-02	0.765
ANGPTL3	1.16 (1.02–1.32)	2.75E-02	0.768
GDF-15	1.13 (1.00–1.28)	4.39E-02	0.762
**Obstructive Physiology N = 832**			
**Baseline clinic model**			**0.753**
sRAGE	1.13 (1.04–1.22)	2.80E-03	0.754
ADIPSIN	1.12 (1.03–1.23)	1.07E-02	0.753
BNP	1.09 (1.02–1.17)	1.25E-02	0.755
CYSTATIN-C	1.11 (1.02–1.21)	1.55E-02	0.757
LEPTIN	1.12 (1.02–1.23)	2.36E-02	0.755
DDP4	0.91 (0.84,0.99)	2.94E-02	0.754

Odds ratio represents difference of protein distribution for cases of obstructive/restrictive physiology in contrast to that for participants with normal PFT. Plasma proteins with nominal p-value<0.05 were displayed in the table.

### Significant linear associations between proteins and lung function for smokers and never-smokers

**Fig 2:**
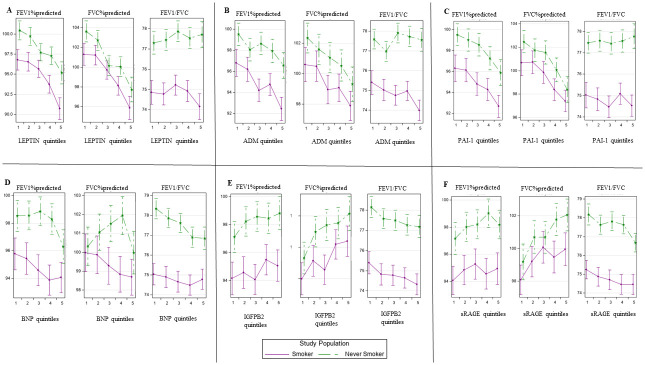
Association of selected proteins (quintiles) with lung function stratified by smoking status. Adjusted least square means of spirometry traits across quintiles of the four protein biomarkers with the greatest inverse effect on FEV_1predicted%_ and two protein biomarkers with positive association on FEV_1%predicted_ and FVC_%predicted_ (A. Leptin; B. ADM; C. PAI-1; D. BNP; E. IGFBP2; F. sRAGE).

In exploratory analyses, we examined the effect of smoking status on the association of proteins with spirometry traits. Fig 2 displays adjusted least square means of spirometry traits across quintiles of the four protein biomarkers with the greatest inverse effect on FEV_1predicted%_ and two protein biomarkers with positive association on FEV_1%predicted_ and FVC_%predicted_, separated by smoking status. The stratified results reveal that spirometry values were consistently lower in smokers that non-smokers, and that most trends for associations of biomarkers with lung function were similar in current or former smokers versus never smokers.

We evaluated potential multiplicative interaction between smoking exposure and protein concentrations (S3 Table). There was a statistically significant interaction of smoking with ADM level for FEV_1%predicted_, FVC_%predicted_, and FEV_1_/FVC (P≤0.05 for all). Specifically, ADM appeared to be associated with greater reduction in FEV_1%predicted_, FVC_%predicted_ and FEV_1_/FVC among smokers in comparison to never-smokers.

### Longitudinal analyses of proteins associated with change in pulmonary function

After excluding individuals with obstructive or restrictive physiology at baseline, a total of 4477 FHS participants with spirometry at the subsequent examination cycle were included in the longitudinal analysis. Three proteins (sRAGE, kallikrein B1 (KLKB1) and APOA1) were associated with longitudinal change in FEV_1%predicted_ (Table 4). Four proteins (sRAGE, KLKB1, APOA-1, and fibrinogen (FBN)) were associated with longitudinal change in FVC_%predicted_ (Table 4). No proteins were associated with change in FEV_1_/FVC.

**Table 4:** Proteins associated with annual change in FEV_1%predicted_, FVC_%predicted_, and/or FEV_1_/FVC.

	ΔFEV_1_/year			ΔFVC/year			Δ FEV_1_/FVC/year		
	Beta*	SE	p-value	Beta	SE	p-value	Beta	SE	p-value
sRAGE	**0.09**	**0.02**	**2.28E-06**	**0.10**	**0.02**	**9.09E-10**	-0.01	0.01	0.49
KLKB1	**-0.07**	**0.02**	**4.70E-05**	**-0.08**	**0.02**	**6.28E-07**	0.01	0.01	0.44
APOA1	**-0.07**	**0.02**	**1.75E-04**	**-0.07**	**0.02**	**2.46E-05**	0.00	0.01	0.96
FBN	-0.05	0.02	2.63E-03	**-0.06**	**0.02**	**5.10E-04**	0.00	0.01	0.95

*Beta coefficient represents change in Δlung function measurement/year per 1-SD change in rank normalized protein. MV model adjusted for age, sex, body mass index (BMI), smoking status (yes = current or former, no = never), pack-years of cigarette smoking, and diabetes mellitus (yes/no).

*Bolded numbers represent statistically significant values meeting Bonferroni corrected p-value threshold: 0.05/71 = 7.04E-04.

**Fig 3:**
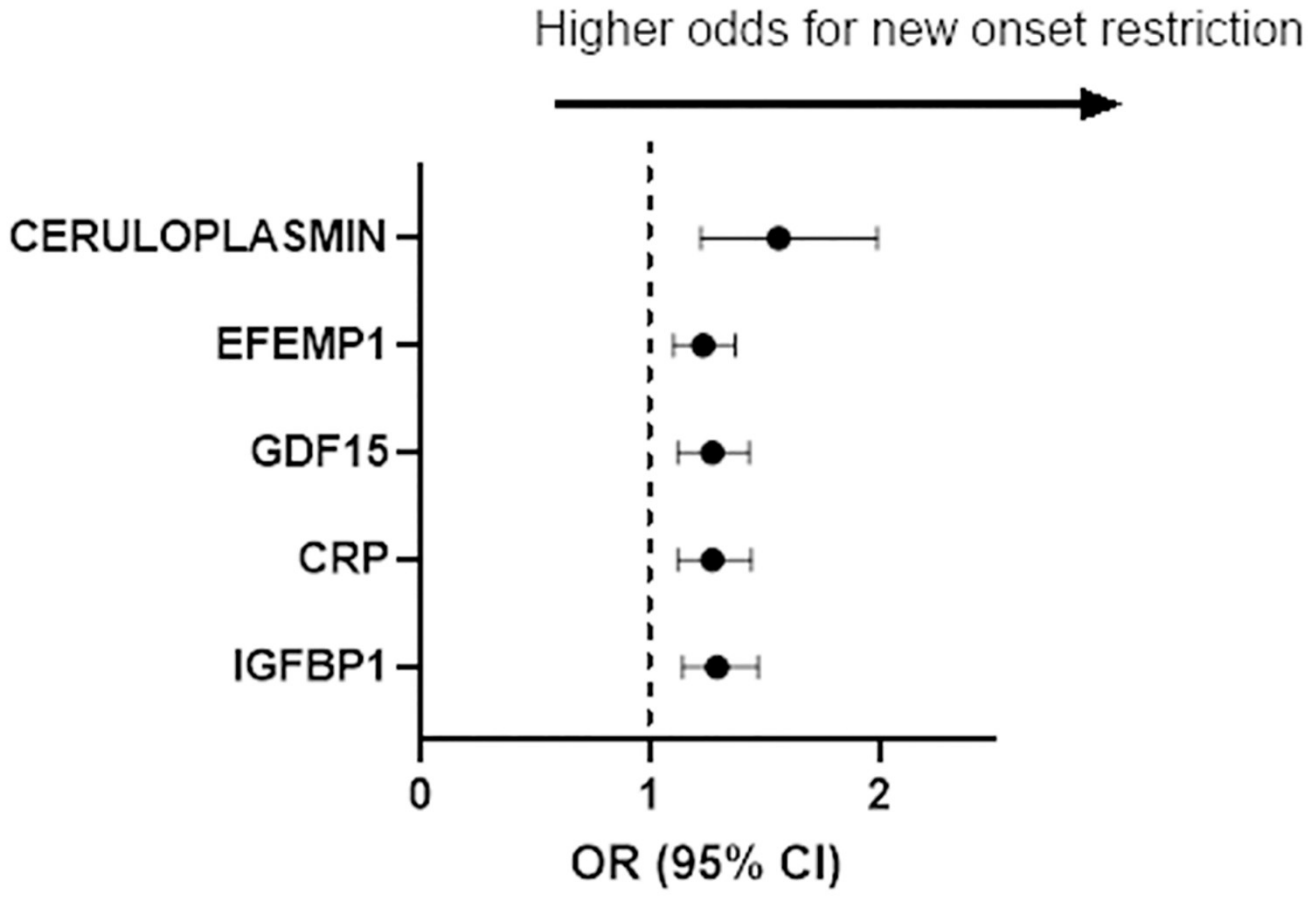
Proteins associated with higher odds of new onset restrictive lung physiology. Ceruloplasmin, EFEMP1, GDF-15, CRP and IGFBP1 had a higher odd of developing restriction as defined by an FVC<80% and FEV_1_/FVC>0.7 when pulmonary function tests were analyzed longitudinally within the cohort.

In secondary analyses, we examined proteins associated with new-onset restrictive and obstructive physiology. Over a mean follow-up of 6 years, 56 individuals developed new-onset restrictive physiology and 206 developed new-onset obstructive physiology. Five proteins were associated with new-onset restrictive physiology: IGFBP1, CRP, GDF-15, epithelial growth factor containing fibulin extracellular matrix protein 1 (EFEMP1), and ceruloplasmin (Fig 3). There were no proteins associated with new-onset obstructive physiology.

## Discussion

**Fig 4:**
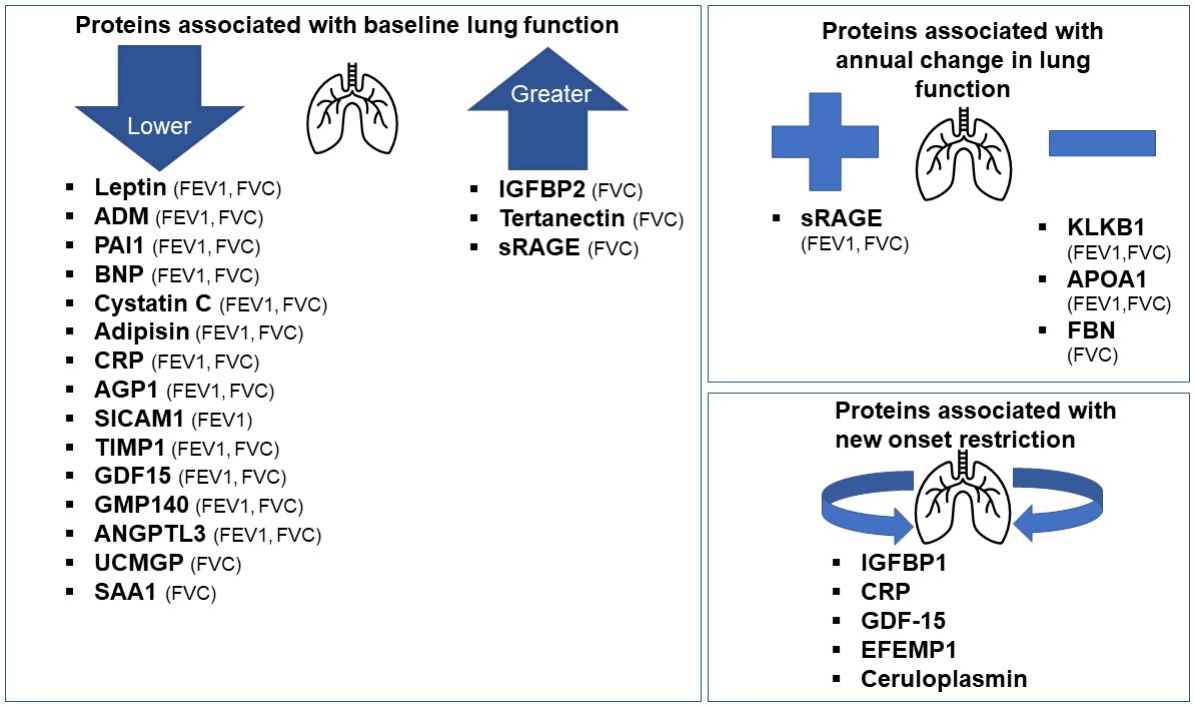


We examined 71 circulating CVD-related proteins for their associations with lung function in nearly 6000 FHS participants. Our main findings are three-fold: first, we identified 18 proteins associated with baseline FEV_1%predicted_ and/or FVC_%predicted_ (Leptin, ADM, PAI1,BNP, Cystatin-C, Adipisin, CRP, AGP1, SICAM1, TIMP1, GDF15, GMP140, ANGPTL3, UCMGP, SAA1, IGFPB2, Tertanectin, SRAGE) (Fig 4). These included adipokines (leptin), markers of inflammation (ADM, PAI-1, CRP, sRAGE), and markers of fibrosis (IGFBP2). Second, we identified four proteins associated with longitudinal changes in FEV_1%predicted_ and/ or FVC_%predicted_ (sRAGE, KLKB1, APOA1, and FBN). Third, five proteins were associated new-onset restrictive physiology (IGFBP1, CRP, GDF-15, EFEMP1, and ceruloplasmin). Taken together, these findings suggest that adipokine-related signaling, inflammation, and fibrosis that are processes known to be associated with CVD also may underlie pulmonary dysfunction.

Lung disease and CVD are inextricably linked and interplay between the pulmonary and cardiac systems affect clinical outcomes [8, 19]. The biological mechanisms that link cardiac and pulmonary disease are not completely understood, but may relate to shared systemic inflammation leading to endothelial dysfunction, tissue dysregulation, and impaired vascular response [20]. While associations between CVD and pulmonary diseases have been well established, there is growing evidence that subtle changes in spirometry indices of lung function, even within the normal range, are predictive of risk for future cardiac disease [21]. In particular, reductions in FEV_1_ and FVC with a preserved FEV_1_/FVC (consistent with restrictive lung changes) in a young ostensibly healthy cohort was reported to be associated with adverse cardiac remodeling, increased left ventricular wall thickness, left ventricular mass, and increased odds of diastolic dysfunction [21, 22]. The association of FVC with increased CVD risk has been demonstrated in the FHS, with early studies reporting a lower FVC to be associated with risk of heart failure and CVD mortality [8]. In addition, in the Jackson Heart Study (JHS) cohort, a restrictive spirometry pattern was associated with increased risk of HF hospitalization and higher pulmonary artery systolic pressure (PASP) [23].

In this context, it is notable that our study findings demonstrate associations of specific CVD-related proteins including leptin, ADM, CRP, cystatin-C, GDF-15, and adipsin with a lower FEV_1_ and FVC, but without a lower FEV_1_/FVC ratio, consistent with restrictive physiology. These proteins in turn have previously been linked by our group to new onset of atherosclerotic CVD, heart failure, CVD-related death, and all-cause mortality (Table 5) [11]. Higher ADM levels are associated with adverse cardiac outcomes and with chronic lung conditions such as asthma or COPD [24]. Higher ADM levels in the setting of cardiopulmonary disease could reflect protective measures to limit the production of pro-inflammatory cytokines as well as help repair endothelial cells [25]. These findings highlight potential shared proteins that may contribute both to CVD and the development of pulmonary disease.

**Table 5:** Proteins associated with baseline lung function and cardiovascular disease outcomes.

	FEV_1_	FVC	FEV_1_/FVC	Atherosclerotic CVD	Heart Failure	CVD mortality	All- Cause Mortality
LEPTIN	↓	↓			↑		
ADM	↓	↓			↑	↑	↑
BNP	↓	↓			↑	↑	↑
CYSTATIN C	↓	↓		↑	↑	↑	↑
CRP	↓	↓			↑	↑	↑
ADIPSIN	↓	↓			↑	↑	↑
sICAM1	↓			↑		↑	↑
GDF-15	↓	↓		↑	↑	↑	↑
AGP-1	↓	↓		↑		↑	↑
TIMP-1	↓	↓		↑	↑	↑	↑
UCMGP	↓	↓				↑	↑
SAA1		↓				↑	↑
IGFBP2		↓				↑	↑
TERTANECTIN		↑				↓	↓
SRAGE		↑	↓				↓

Given the shared role of cigarette smoking as a risk factor for CVD and pulmonary disease, we examined the interaction of smoking with CVD proteins. ADM demonstrated a significant smoking interaction across the lung function parameters; smokers with the highest level of ADM demonstrated reduced lung function. ADM has been shown to be elevated in COPD patients in comparison to healthy controls and it was reported to independently predict intensive care unit (ICU) admission during COPD exacerbation [26]. In addition, pro-ADM has been shown to be an independent predictor of all-cause mortality in stable COPD patients and has been shown to improve the accuracy of 1-year and 2-year mortality prediction when added to the BODE (body mass index, airflow obstruction, dyspnea and exercise capacity) index [27]. Cigarette smoke and aryl hydrocarbon receptor (AHR) activating ligands have been shown to upregulate ADM expression *in vitro* and *in vivo* [28]. Our results further confirm the inverse association of ADM with lung function when considered in the context of cigarette smoke exposure.

The majority of protein biomarkers were associated with reduced lung function; however, sRAGE, tetranectin, and IGFBP2 were associated with preserved lung function as reflected by higher baseline FVC_%predicted_. Higher sRAGE levels have previously been associated with higher FVC, total lung capacity (TLC), and diffusion (DLCO) suggesting beneficial effects on the lungs [29–31]. sRAGE has been proposed to act on advanced glycation end-products (AGEs) to inhibit their ability to prevent wound healing and destroy the extracellular matrix [29]. Tetranectin and sRAGE have also been reported to be associated with favorable cardiac features. In particular, lower serum tetranectin levels have been associated with higher prevalence of coronary artery disease and have been recorded in patients with acute myocardial infarction [32, 33]. Tetranectin engages in thrombolysis by binding to fibrin and converting plasminogen to plasmin, therefore lower levels of tetranectin could lead to higher rates of thrombosis [33]. Tetranectin and sRAGE have been reported to be associated with lower all-cause mortality risk and our study further highlights their potential protective effects with regard to the lungs [11, 34].

Similar to tetranectin and sRAGE, IGFBP2 demonstrated a positive association with FEV_1_ and FVC in cross-sectional analyses. Guiot et al. demonstrated higher serum IGFBP2 levels in patients with idiopathic pulmonary fibrosis (IPF); however, when gene expression profiles of the lung fibroblast were examined in IPF and control patients, IGFBP2 was >10 higher in the controls [35, 36]. IGFBP2 has been reported to be associated with adverse cardiac outcomes including CVD death and reduced LVEF [11, 37]. IGFBP2 may have protective roles in both the lungs and heart by downregulating insulin growth factor (IGF) in the lung and reducing fibroblast formation, as well as limiting myoblast formation in the heart [36, 37]. Given that IGFBP2 serum levels decrease in response to initiation of anti-fibrotic medication in IPF patients, it is possible that serum IGFBP2 is upregulated in response to inflammation or damage within the lungs [36].

In addition to identifying 18 proteins associated with FEV_1%predicted_ and/or FVC_%predicted_ with little effect on the FEV_1_/FVC ratio, we identified five proteins (IGFBP1, CRP, GDF-15, EFEMP1, and ceruloplasmin) associated with new-onset restrictive physiology (after adjusting for BMI) [38]. GDF-15 is an epithelial stress marker that is elevated in patients with idiopathic pulmonary fibrosis (IPF), a predictor of more severe disease and worse outcomes in IPF and associated with a greater odd of developing interstitial lung abnormalities (ILA) in both the FHS and COPDGene cohorts [38, 39]. Higher levels of baseline GDF-15 have also been associated with a more rapid decline in FEV_1_ over the span of 5 years in a community based sample [40]. Elevated levels of GDF-15 have been demonstrated to be an independent predictor of heart failure related rehospitalization as well as death in patients with both diastolic and systolic heart failure after adjusting for troponin and BNP levels [41]. In addition, elevated GDF-15 have been associated with higher right atrial pressures and adverse outcomes in patient with idiopathic pulmonary arterial hypertension (PAH) [42]. While the association of GDF-15 with lung fibrosis, heart failure, and PAH has previously been established, our results demonstrate a novel association of EFEMP1 with new onset of restrictive lung physiology [38]. EFEMP1 is known to play a role in cell-to-cell and cell-to-matrix communication and inhibits cell growth [43]. An elevated EFEMP1 has previously been demonstrated to be associated with a greater odds of heart failure, CVD mortality, and all-cause mortality, suggesting that it may have roles in the development or progression of cardiac and lung diseases [11]. Of note, the association of GDF-15 and EFEMP1 with new onset restrictive physiology was independent of BMI.

Our study has several limitations worth noting. We utilized a panel of 71 high value CVD plasma proteins to assess their associations with lung function. These proteins represent an incomplete proteomic fingerprint of lung disease. In addition, this was an observational study, which limits inferences of causality and biological mechanisms underlying lung disease. Furthermore, we defined restrictive and obstructive physiology based solely upon spirometry measures. Body-plethysmography may provide a more specific test for restrictive pulmonary disease as a decrease in FVC may be the result of increased dead space. Finally, the participants in this study were predominantly white, limiting potential generalizability of the study findings to other racial/ethnic groups.

In conclusion, our findings highlight several potential shared proteins between lung function and CVD. Specifically, we identified 18 proteins associated with baseline and four proteins associated with longitudinal changes in FEV_1%predicted_ and/or FVC_%predicted_. Many of the proteins demonstrate patterns of association with lung function that are suggestive of restrictive lung physiology. We identified five proteins associated with new onset restrictive lung physiology. The proteins identified include markers of inflammation, adiposity, and fibrosis reflecting proteins that may contribute to lung function and either directly or indirectly affect cardiac function. Further studies are needed to explore the mechanisms underlying shared proteins involved in lung and cardiac diseases.

## Supporting information

S1 Table Clinical characteristics of 5777 FHS participants. (DOCX)

S2 Table Association of 71 cardiovascular disease related plasma proteins with baseline FEV_1%predicted_, FVC_%predicted_, and/or FEV_1_/FVC. Beta coefficient represents change in lung function measurement per 1-SD change in rank normalized protein. MV model adjusted for age, sex, body mass index (BMI), smoking status (current, former, never), pack-years of cigarette smoking, and diabetes mellitus (yes/no).
(DOCX)

S3 Table Association of selected proteins with lung function stratified by smoking status. Beta coefficient represents correlations between lung function and protein distribution. MV model adjusted for age, sex, body mass index (BMI), smoking status (current, former, never), pack-years of cigarette smoking, and diabetes mellitus (yes/no). Interaction term (smoking status*biomarker) was evaluated in total sample.
(DOCX)

## Abbreviations

CAD: Coronary Artery Disease
CVD: Cardiovascular disease
FEV_1_: Forced Expiratory Volume in one second
FVC: Forced Vital Capacity
