# Supplementary material for: Association of 71 cardiovascular disease-related plasma proteins with pulmonary function in the community
Source: PLoS One. 2022 Apr 7;17(4):e0266523. doi: 10.1371/journal.pone.0266523 (PMC8989231; doi:10.1371/journal.pone.0266523)
Supplement: S1 Table — (DOCX) [file pone.0266523.s001.docx]

**S1 Table. Clinical characteristics of 5777 FHS participants.**

|  | ALL (N=5777) | Offspring (N=2217) | Gen 3 (N=3560) |
| --- | --- | --- | --- |
| Age, years, mean (SD) | 48 (13) | 60 (9) | 40 (9) |
| Women, n (%) | 3089 (54) | 1217 (55) | 1872 (53) |
| Current smoker, n (%) | 808 (14) | 267 (12) | 541 (15) |
| Former smoker, n (%) | 2125 (37) | 1150 (52) | 975 (27) |
| Pack-years smoking | 10 (16) | 16 (21) | 6 (11) |
| Body-mass-index, kg/m^2^ | 27.4 (5.4) | 28.1 (5.3) | 26.9 (5.5) |
| History of Cardiovascular Disease, n (%) | 241 (4) | 217 (10) | 24 (1) |
| Diabetes Mellitus, n (%) | 326 (6) | 228 (10) | 98 (3) |
| FEV_1_  _% Predicted_ (SD) | 97 (14) | 92 (15) | 100 (12) |
| FVC _% Predicted_ (SD) | 101 (12) | 97 (13) | 103 (11) |
| FEV_1_/FVC _% Predicted_  (SD) | 96 (8) | 95 (9) | 96 (7) |
| Obstructive physiology category 1, n (%) | 489 (8) | 278 (13) | 211 (6) |
| Obstructive physiology category 2, n (%) | 343 (6) | 259 (12) | 84 (2) |
| Restrictive physiology, n (%) | 201 (3) | 135 (6) | 66 (2) |

Mean (SD), n (%)
