# Supplementary material for: Association of 71 cardiovascular disease-related plasma proteins with pulmonary function in the community
Source: PLoS One. 2022 Apr 7;17(4):e0266523. doi: 10.1371/journal.pone.0266523 (PMC8989231; doi:10.1371/journal.pone.0266523)
Supplement: S2 Table — Beta coefficient represents change in lung function measurement per 1-SD change in rank normalized protein. MV model adjusted for age, sex, body mass index (BMI), smoking status (current, former, never), pack-years of cigarette smoking, and diabetes mellitus (yes/no). (DOCX) [file pone.0266523.s002.docx]

**S2 Table.** **Association of 71 Cardiovascular Disease Related Plasma Proteins with baseline FEV_1%predicted_, FVC_%predicted_, and/or FEV_1_/FVC.**

|  |  | FEV_1%predicted_ | | | FVC_%predicted_ | | | FEV_1_/FVC (%) | | |
| --- | --- | --- | --- | --- | --- | --- | --- | --- | --- | --- |
| Abbreviation | Protein name | beta | se | p-value | beta | se | p-value | beta | se | p-value |
| A1M | Alpha-1-microglobulin (A1M) | 0.17 | 0.18 | 3.34E-01 | 0.38 | 0.16 | 1.68E-02 | -0.14 | 0.08 | 8.34E-02 |
| ADAM15 | Disintegrin and metalloproteinase domain-containing protein 15 (ADAM15) | 0.06 | 0.17 | 7.24E-01 | 0.08 | 0.15 | 5.81E-01 | -0.01 | 0.08 | 8.72E-01 |
| Adipsin | Adipsin | -0.92 | 0.19 | 1.26E-06 | -0.73 | 0.17 | 1.70E-05 | -0.22 | 0.09 | 1.17E-02 |
| ADM | Adrenomedullin | -1.20 | 0.20 | 1.98E-09 | -1.05 | 0.18 | 3.58E-09 | -0.14 | 0.09 | 1.33E-01 |
| AGP1 | Alpha-1-acid glycoprotein 1 | -0.66 | 0.18 | 1.78E-04 | -0.62 | 0.16 | 7.81E-05 | -0.06 | 0.08 | 4.70E-01 |
| ANGPTL3 | Angiopoietin-like 3 | -0.62 | 0.18 | 6.72E-04 | -0.66 | 0.16 | 5.13E-05 | 0.01 | 0.09 | 9.16E-01 |
| APOA1 | Apolipoprotein A-1 | -0.03 | 0.18 | 8.87E-01 | 0.19 | 0.16 | 2.29E-01 | -0.16 | 0.08 | 5.22E-02 |
| APOB | Apolipoprotein B | -0.12 | 0.18 | 4.88E-01 | -0.33 | 0.16 | 3.50E-02 | 0.16 | 0.08 | 5.12E-02 |
| B2M | Beta-2-microglobulin | -0.56 | 0.19 | 2.89E-03 | -0.48 | 0.17 | 4.23E-03 | -0.09 | 0.09 | 3.33E-01 |
| BCHE | Butyrylcholine esterase (BCHE) | -0.37 | 0.17 | 3.33E-02 | -0.35 | 0.16 | 2.46E-02 | -0.04 | 0.08 | 5.92E-01 |
| BIKUNIN | AMBP-bikunin (BIKUNIN) | 0.22 | 0.17 | 1.95E-01 | 0.34 | 0.15 | 2.42E-02 | -0.08 | 0.08 | 3.32E-01 |
| BNP | N-terminal prohormone of brain natriuretic peptide | -1.01 | 0.18 | 2.57E-08 | -0.74 | 0.16 | 4.32E-06 | -0.21 | 0.08 | 1.29E-02 |
| C2 | Complement C2 | -0.35 | 0.18 | 4.54E-02 | -0.44 | 0.16 | 4.63E-03 | 0.07 | 0.08 | 3.70E-01 |
| CD14 | Monocyte differentiation antigen CD14 | -0.09 | 0.17 | 5.83E-01 | 0.07 | 0.15 | 6.35E-01 | -0.13 | 0.08 | 9.71E-02 |
| CD163 | Scavenger receptor cysteine-rich type 1 protein (M130/CD163) | -0.09 | 0.17 | 5.84E-01 | -0.08 | 0.15 | 5.91E-01 | 0.00 | 0.08 | 9.93E-01 |
| CD40L | Soluble CD40 ligand | 0.05 | 0.17 | 7.86E-01 | -0.07 | 0.15 | 6.33E-01 | 0.10 | 0.08 | 2.03E-01 |
| CD56 | Neural cell adhesion molecule (CD56) | -0.17 | 0.19 | 3.60E-01 | -0.23 | 0.17 | 1.84E-01 | 0.02 | 0.09 | 7.99E-01 |
| CD5L | CD5 antigen-like(CD5L) | -0.30 | 0.17 | 8.16E-02 | -0.32 | 0.15 | 4.06E-02 | -0.02 | 0.08 | 8.41E-01 |
| CDH13 | Cadherin-13 (CDH13) | 0.01 | 0.17 | 9.63E-01 | 0.04 | 0.15 | 7.62E-01 | -0.03 | 0.08 | 6.58E-01 |
| Ceruloplasmin | Ceruloplasmin | -0.42 | 0.19 | 2.51E-02 | -0.52 | 0.17 | 1.65E-03 | 0.11 | 0.09 | 2.08E-01 |
| Clusterin | Clusterin | -0.02 | 0.17 | 9.23E-01 | 0.03 | 0.15 | 8.27E-01 | -0.04 | 0.08 | 6.28E-01 |
| CNTN1 | Contactin 1 | -0.04 | 0.18 | 8.05E-01 | 0.00 | 0.16 | 9.83E-01 | -0.03 | 0.08 | 7.21E-01 |
| COL18aA | Collagen, type XVIII, alpha 1 | -0.08 | 0.17 | 6.37E-01 | -0.11 | 0.15 | 4.63E-01 | 0.02 | 0.08 | 8.12E-01 |
| CRP | C-Reactive Protein | -0.84 | 0.17 | 1.68E-06 | -1.06 | 0.16 | 9.25E-12 | 0.18 | 0.08 | 2.50E-02 |
| CXCL16 | Chemokine (C-X-C motif) ligand 16 | -0.50 | 0.17 | 3.69E-03 | -0.49 | 0.15 | 1.41E-03 | -0.01 | 0.08 | 8.86E-01 |
| Cystatin C | Cystatin-C | -0.97 | 0.19 | 2.56E-07 | -0.75 | 0.17 | 7.90E-06 | -0.22 | 0.09 | 1.26E-02 |
| DPP4 | Dipeptidyl-peptidase 4 (DPP4) | 0.05 | 0.17 | 7.57E-01 | 0.09 | 0.15 | 5.36E-01 | -0.02 | 0.08 | 7.78E-01 |
| EFEMP1 | EGF containing fibulin-like extracellular matrix protein 1 (EFEMP1) | -0.27 | 0.19 | 1.66E-01 | -0.08 | 0.17 | 6.40E-01 | -0.14 | 0.09 | 1.23E-01 |
| FBN | Fibrinogen (FBN) | -0.33 | 0.18 | 5.78E-02 | -0.43 | 0.16 | 6.60E-03 | 0.06 | 0.08 | 4.54E-01 |
| FGF23 | Fibroblast growth factor 23 | -0.17 | 0.17 | 2.93E-01 | -0.16 | 0.15 | 2.82E-01 | -0.02 | 0.08 | 7.49E-01 |
| GADPH | Glyceraldehyde 3-phosphate dehydrogenase | 0.24 | 0.17 | 1.48E-01 | 0.16 | 0.15 | 2.95E-01 | 0.07 | 0.08 | 3.43E-01 |
| GDF-15 | Growth differentiation factor 15 | -0.75 | 0.20 | 2.53E-04 | -0.77 | 0.18 | 2.57E-05 | 0.01 | 0.10 | 9.02E-01 |
| GMP-140 | Granule membrane protein 140 (P-selectin) | -0.60 | 0.17 | 5.67E-04 | -0.60 | 0.16 | 1.27E-04 | -0.04 | 0.08 | 6.28E-01 |
| GP5 | Glycoprotein V (platelet) | -0.15 | 0.17 | 3.74E-01 | -0.19 | 0.15 | 2.02E-01 | 0.03 | 0.08 | 6.76E-01 |
| GRN | Granulin | -0.07 | 0.17 | 6.76E-01 | -0.21 | 0.15 | 1.57E-01 | 0.12 | 0.08 | 1.24E-01 |
| Hemopexin | Hemopexin | -0.17 | 0.18 | 3.34E-01 | -0.32 | 0.16 | 4.57E-02 | 0.12 | 0.08 | 1.43E-01 |
| IGF1 | Insulin-like growth factor 1 | 0.28 | 0.19 | 1.38E-01 | 0.26 | 0.17 | 1.10E-01 | 0.04 | 0.09 | 6.45E-01 |
| IGFBP3 | Insulin-like growth factor-binding protein 3 | -0.17 | 0.17 | 3.18E-01 | -0.11 | 0.15 | 4.61E-01 | -0.08 | 0.08 | 2.97E-01 |
| IGFBP1 | Insulin-like growth factor-binding protein 1 | -0.13 | 0.18 | 4.74E-01 | -0.06 | 0.16 | 7.06E-01 | -0.01 | 0.08 | 8.75E-01 |
| IGFBP2 | Insulin-like growth factor binding protein 2 (IGFBP2) | 0.50 | 0.19 | 7.01E-03 | 0.90 | 0.17 | 5.63E-08 | -0.29 | 0.09 | 8.50E-04 |
| KLKB1 | Plasma kallikrein (KLKB1) | -0.05 | 0.17 | 7.68E-01 | 0.03 | 0.15 | 8.29E-01 | -0.06 | 0.08 | 4.54E-01 |
| LDLR | Low density lipoprotein receptor (LDLR) | -0.02 | 0.17 | 8.91E-01 | -0.03 | 0.15 | 8.21E-01 | 0.02 | 0.08 | 8.27E-01 |
| Leptin | Leptin | -1.36 | 0.22 | 5.85E-10 | -1.22 | 0.20 | 5.99E-10 | -0.19 | 0.10 | 6.55E-02 |
| Leptin-r | Leptin receptor | -0.03 | 0.17 | 8.53E-01 | -0.05 | 0.15 | 7.61E-01 | 0.02 | 0.08 | 8.03E-01 |
| LPA | Lipoprotein(a) | -0.18 | 0.17 | 2.98E-01 | -0.06 | 0.15 | 6.97E-01 | -0.13 | 0.08 | 1.17E-01 |
| MCAM | Melanoma cell adhesion molecule (MCAM) | -0.31 | 0.18 | 7.41E-02 | -0.05 | 0.16 | 7.34E-01 | -0.21 | 0.08 | 1.23E-02 |
| MCP1 | Monocyte chemotactic molecule 1 | -0.39 | 0.17 | 2.06E-02 | -0.39 | 0.15 | 1.08E-02 | -0.02 | 0.08 | 8.42E-01 |
| MMP9 | Matrix metallopeptidase 9 | -0.35 | 0.17 | 3.76E-02 | -0.21 | 0.15 | 1.60E-01 | -0.11 | 0.08 | 1.44E-01 |
| MMP8 | Matrix metallopeptidase 8 (MMP8) | 0.14 | 0.17 | 4.23E-01 | 0.12 | 0.15 | 4.22E-01 | 0.00 | 0.08 | 9.70E-01 |
| MPO | Myeloperoxidase | -0.36 | 0.17 | 3.71E-02 | -0.41 | 0.15 | 6.70E-03 | 0.03 | 0.08 | 6.94E-01 |
| Myoglobin | Myoglobin | 0.19 | 0.17 | 2.83E-01 | 0.22 | 0.16 | 1.63E-01 | -0.04 | 0.08 | 6.00E-01 |
| Notch1 | Translocation associated notch homolog (Notch1) | -0.02 | 0.17 | 9.15E-01 | -0.11 | 0.15 | 4.60E-01 | 0.08 | 0.08 | 2.96E-01 |
| NRCAM | Neuronal cell adhesion molecule (NRCAM) | 0.01 | 0.17 | 9.69E-01 | 0.08 | 0.15 | 5.79E-01 | -0.05 | 0.08 | 4.95E-01 |
| Osteo | Osteocalcin | 0.16 | 0.17 | 3.51E-01 | 0.12 | 0.15 | 4.35E-01 | 0.02 | 0.08 | 8.19E-01 |
| PAI1 | Plasminogen activator inhibitor 1 | -1.06 | 0.19 | 1.25E-08 | -1.09 | 0.17 | 5.03E-11 | -0.01 | 0.09 | 8.74E-01 |
| PMP2 | Myelin P2 protein (PMP2) | 0.07 | 0.17 | 6.58E-01 | 0.13 | 0.15 | 3.76E-01 | -0.04 | 0.08 | 6.47E-01 |
| PON1 | Serum paraoxonase/arylesterase 1 | -0.30 | 0.17 | 8.96E-02 | -0.18 | 0.16 | 2.36E-01 | -0.09 | 0.08 | 2.74E-01 |
| PPBP | Pro-platelet basic protein (PPBP) | -0.01 | 0.17 | 9.42E-01 | -0.09 | 0.15 | 5.39E-01 | 0.06 | 0.08 | 4.25E-01 |
| PZI | Protein Z-dependent protease inhibitor | -0.15 | 0.17 | 3.76E-01 | -0.15 | 0.15 | 3.10E-01 | 0.00 | 0.08 | 9.65E-01 |
| REG1A | Lithostathine-1-alpha | -0.13 | 0.17 | 4.55E-01 | 0.03 | 0.15 | 8.42E-01 | -0.14 | 0.08 | 8.59E-02 |
| Resistin | Resistin | -0.08 | 0.17 | 6.30E-01 | -0.06 | 0.15 | 7.14E-01 | -0.01 | 0.08 | 9.38E-01 |
| SAA1 | Serum amyloid A1 | -0.55 | 0.17 | 1.24E-03 | -0.75 | 0.15 | 5.78E-07 | 0.17 | 0.08 | 3.51E-02 |
| SDF1 | Stromal cell-derived factor 1 | -0.02 | 0.17 | 9.04E-01 | -0.05 | 0.15 | 7.49E-01 | 0.01 | 0.08 | 9.04E-01 |
| sGP130 | Interleukin-6 receptor beta | -0.45 | 0.17 | 9.63E-03 | -0.34 | 0.15 | 2.58E-02 | -0.11 | 0.08 | 1.89E-01 |
| sICAM1 | Intercellular adhesion molecule 1 | -0.64 | 0.17 | 2.19E-04 | -0.47 | 0.15 | 2.08E-03 | -0.14 | 0.08 | 8.12E-02 |
| sRAGE | Receptor for advanced glycation end products | 0.24 | 0.18 | 1.67E-01 | 0.70 | 0.16 | 8.86E-06 | -0.35 | 0.08 | 2.41E-05 |
| Tetranectin | Tetranectin | 0.28 | 0.17 | 9.12E-02 | 0.54 | 0.15 | 2.91E-04 | -0.22 | 0.08 | 5.01E-03 |
| TIMP1 | Tissue inhibitor of metalloproteinases 1 | -0.71 | 0.19 | 2.35E-04 | -0.63 | 0.17 | 2.72E-04 | -0.11 | 0.09 | 2.23E-01 |
| TSC22D3 | TSC22 domain family, member 3 (TSC22D3) | 0.05 | 0.17 | 7.83E-01 | 0.04 | 0.15 | 8.11E-01 | 0.01 | 0.08 | 9.37E-01 |
| UCMGP | Uncarboxylated matrix gla protein (UCMGP) | -0.60 | 0.19 | 1.71E-03 | -0.62 | 0.17 | 2.38E-04 | -0.01 | 0.09 | 8.68E-01 |
| VEGF | Vascular endothelial growth factor | -0.35 | 0.17 | 3.34E-02 | -0.30 | 0.15 | 4.28E-02 | -0.03 | 0.08 | 7.09E-01 |

Beta coefficient represents change in lung function measurement per 1-SD change in rank normalized protein. MV model adjusted for age, sex, body mass index (BMI), smoking status (current, former, never), pack-years of cigarette smoking, and diabetes mellitus (yes/no).
