# Supplementary material for: Association of 71 cardiovascular disease-related plasma proteins with pulmonary function in the community
Source: PLoS One. 2022 Apr 7;17(4):e0266523. doi: 10.1371/journal.pone.0266523 (PMC8989231; doi:10.1371/journal.pone.0266523)
Supplement: S3 Table — Beta coefficient represents correlations between lung function and protein distribution. MV model adjusted for age, sex, body mass index (BMI), smoking status (current, former, never), pack-years of cigarette smoking, and diabetes mellitus (yes/no). Interaction term (smoking status*biomarker) was evaluated in total sample. (DOCX) [file pone.0266523.s003.docx]

**S3 Table. Association of selected proteins with lung function stratified by smoking status.**

|  | FEV_1%predicted_ | | | | | FVC_%predicted_ | | | | | FEV_1_/FVC | | | | |
| --- | --- | --- | --- | --- | --- | --- | --- | --- | --- | --- | --- | --- | --- | --- | --- |
|  | Smoker | | Non-Smoker | | **P_interaction_** | Smoker | | Non-Smoker | | **P_interaction_** | Smoker | | Non-Smoker | | **P_interaction_** |
|  | **Beta** | **SE** | **Beta** | **SE** |  | **Beta** | **SE** | **Beta** | **SE** |  | **Beta** | **SE** | **Beta** | **SE** |  |
| LEPTIN | -1.29 | 0.32 | -1.59 | 0.30 | 0.57 | -0.98 | 0.28 | -1.60 | 0.28 | 0.69 | -0.33 | 0.15 | -0.04 | 0.14 | 0.46 |
| ADM | -1.54 | 0.29 | -0.88 | 0.27 | 1.74E-03 | -1.12 | 0.25 | -1.03 | 0.25 | 0.04 | -0.38 | 0.14 | 0.11 | 0.13 | 0.01 |
| PAI-1 | -1.16 | 0.27 | -1.04 | 0.26 | 0.99 | -1.10 | 0.23 | -1.26 | 0.24 | 0.99 | -0.11 | 0.12 | 0.16 | 0.12 | 0.83 |
| BNP | -0.96 | 0.26 | -1.04 | 0.25 | 0.65 | -0.87 | 0.23 | -0.54 | 0.23 | 0.2 | -0.05 | 0.12 | -0.42 | 0.12 | 0.2 |
| IGFBP2 | 0.41 | 0.27 | 0.50 | 0.26 | 0.33 | 0.93 | 0.23 | 0.78 | 0.24 | 0.75 | -0.37 | 0.13 | -0.22 | 0.12 | 0.05 |
| sRAGE | 0.24 | 0.26 | 0.25 | 0.24 | 0.56 | 0.59 | 0.22 | 0.84 | 0.22 | 0.52 | -0.26 | 0.12 | -0.47 | 0.11 | 0.99 |

Beta coefficient represents correlations between lung function and protein distribution. MV model adjusted for age, sex, body mass index (BMI), smoking status (current, former, never), pack-years of cigarette smoking, and diabetes mellitus (yes/no). Interaction term (smoking status*biomarker) was evaluated in total sample.
